# Proteomic analysis reveals early pathological defects in corticospinal motor neurons of a spastin model of hereditary spastic paraplegia, which are improved by NU-9 treatment

**DOI:** 10.1016/j.nbd.2026.107494

**Published:** 2026-06-19

**Authors:** Mukesh Gautam, Mercedes Priego, Christopher Quintanilla, Omar Kashow, Byoung-Kyu Cho, Young Ah Goo, Richard B. Silverman, Gerardo Morfini, Peter W. Baas, P. Hande Ozdinler

**Affiliations:** a Department of Neurology, Northwestern University Feinberg School of Medicine, Chicago, IL 60611, USA; b Department of Anatomy and Cell Biology, University of Illinois, Chicago, IL, USA; c Mass Spectrometry Technology Access Center at the McDonnell Genome Institute, Washington University School of Medicine, St. Louis, MO, USA; d Department of Biochemistry and Molecular Biophysics, Washington University School of Medicine, St. Louis, MO, USA; e Department of Genetics, Washington University School of Medicine, St. Louis, MO, USA; f Department of Chemistry and Department of Molecular Biosciences, Northwestern University, 2145 Sheridan Road, Evanston, IL, USA; g Department of Pharmacology, Feinberg School of Medicine, Chicago, IL 60611, USA; h Chemistry of Life Processes Institute, Northwestern University, Evanston, IL 60208, USA; i Department of Neurobiology and Anatomy, Drexel University College of Medicine, Philadelphia, PA, USA; j Les Turner ALS Center, Northwestern University, Feinberg School of Medicine, Chicago, IL 60611, USA; k Mesulam Institute for Cognitive Neurology and Alzheimer's Disease, Northwestern University, Feinberg School of Medicine, Chicago, IL 60611, USA

**Keywords:** Spastin, HSP, CSMN, Upper motor neurons, NU-9

## Abstract

Upper motor neuron (UMN) degeneration is a characteristic feature of hereditary spastic paraplegia (HSP), a genetically heterogeneous heritable neurodegenerative disorder resulting from mutations in over ninety genes. The mutations in the *SPAST* gene, which encodes the microtubule-severing protein spastin, are responsible for about 40% of all HSP cases. To date, the cellular and molecular mechanisms linking mutant spastin protein to UMN vulnerability in HSP patients remain unknown and there are no disease modifying therapies. To address this knowledge gap, we isolated pure populations of corticospinal motor neurons (CSMN; a.k.a. UMN in mice) from SPAST^C448Y^-UeGFP reporter mice at two pre-symptomatic time points and performed bottom-up proteomic analyses to reveal changes in their proteome that informs the underlying causes of their initial vulnerability. We find dynamic changes in their proteome and that limitations with cytoarchitectural integrity and stability of key organelles contribute to their neuronal vulnerability. Since the compound **NU-9** was shown to improve similar cellular problems in CSMN that are diseased due to misfolded SOD1 toxicity and TDP-43 pathology, we further investigated its effect on the well-established pathological features of HSP that are recapitulated in the SPAST^C448Y^ mice. We find that **NU-9** treatment (100 mg/kg, for 100 days) significantly prevented degeneration of corticospinal axons, restored the integrity of mitochondria and endoplasmic reticulum, and reduced the presence of electron-dense accumulations in the CSMN of SPAST^C448Y^ mice.

## 1. Introduction

Upper motor neurons (UMNs), also known as corticospinal motor neurons (CSMN), are well-established for their involvement in the initiation and modulation of voluntary movement (Georgopoulos et al., 1986). They are primarily affected in a wide variety of motor neuron diseases, including hereditary spastic paraplegia (HSP; (Blackstone, 2012), primary lateral sclerosis (PLS; (Fullam and Statland, 2021) and amyotrophic lateral sclerosis (ALS; (Brown Jr. and Robberecht, 2001). Even though UMNs degenerate in all these diseases, it remains unclear whether UMN vulnerability and progressive degeneration occur due to similar or distinct mechanisms. Revealing this information is critically important for understanding the cellular basis of UMN vulnerability in different diseases, and for developing effective treatment strategies.

Obtaining reliable cellular data from whole brain extract is not possible. Bringing a mechanistic insight onto cellular events that go awry in distinct neuron populations of the brain is challenging, especially when these neurons account for a small percentage in the brain. UMNs are present only in layer 5 of the motor cortex and they make up less than 1% of the cerebral cortex (Rivara et al., 2003). Yet, their degeneration is the characteristic feature of HSP, PLS and ALS (Calma et al., 2024; Chan et al., 2003; Finegan et al., 2020; Mania-Paris et al., 2025; Ozdinler et al., 2020). Thus, they are clinically important neuron populations, and it is important to reveal the underlying mechanisms that contribute to their selective vulnerability.

To date, mutations in more than 90 genes have been linked to HSP (Elsayed et al., 2021; Mackay-Sim, 2021), but mutations in the *SPAST* gene are observed in about 40% of all HSP patients (Fink, 2003), making it a key gene of interest to study mechanisms underlying CSMN vulnerability in HSP. Spastin is a microtubule-severing protein involved in the regulation of microtubule dynamics and the integrity of key cellular organelles such as mitochondria and ER (Errico et al., 2002; Evans et al., 2005; Raby et al., 2024; Roll-Mecak and Vale, 2005; Vajente et al., 2019). SPAST^C448Y^ mice, which express exogenous human *SPAST* gene with the HSP-linked *C448Y* mutation was developed by the Baas group (Qiang et al., 2019) and have been shown to recapitulate several key clinical features of HSP (Deluca et al., 2004; Fink, 2013; Piermarini et al., 2022), making it an ideal model.

To shift focus from mice to diseased neurons, we utilized UCHL1-eGFP mice, in which CSMN are fluorescently labeled (Yasvoina et al., 2013). Crossing SPAST^C448Y^ with UCHL1-eGFP generated SPAST^C448Y^-UeGFP mice, an HSP disease model in which CSMN are eGFP+ (green fluorescent). CSMN can then be isolated as a pure neuron population from the complex structure of the brain, via FACS (fluorescence activated cell sorting) mediated purification strategies, as previously reported for other disease models (Gautam et al., 2019a; Gautam et al., 2016). In this study, pure populations of CSMN are isolated from SPAST^C448Y^-UeGFP mice at two different and early time points, helping us reveal the cellular and molecular basis of the initial cellular events that lead to their vulnerability.

Utilization of proteomic approaches to understand the underlying disease mechanisms is gaining attention (Aebersold and Mann, 2016; Bai et al., 2021; Gautam et al., 2026; Nementzik et al., 2025). Here, we performed bottom-up proteomics on CSMN isolated from SPAST^C448Y^-UeGFP mice to reveal the presence of key proteins in these vulnerable neurons with respect to healthy controls, and to unveil the dynamic changes that that take place in them, with a temporal resolution.

Failure to maintain Rho GTPase dynamics and cytoarchitectural integrity, a decrease in the levels of microautophagy and an attempt to activate the CLEAR signaling pathway emerge as initial key cellular events. Defects at the site of mitochondria and the axon become more prominent with time. These findings suggest that different sets of proteins could become increasingly present or absent at different time points in CSMN that are destined to become diseased. Therefore, the cellular events and canonical pathways, which ultimately contribute to CSMN degeneration in the most common form of HSP can be better understood when pure populations of CSMN are interrogated by proteomic approaches.

**NU-9** was recently identified as a small molecule that can improve the health of CSMN that are diseased due to misfolded SOD1 toxicity and TDP-43 pathology (Genc et al., 2021). The cellular events that were primarily involved in their vulnerability were mostly related to protein accumulation, mitochondrial and cytoarchitectural impairment, especially at the site of the apical dendrites. Albeit ALS and HSP are different UMN diseases, there are common cellular pathologies. **NU-9** treatment improved axon outgrowth in vitro (Genc et al., 2022) and helped improve cytoarchitectural stability at the site of apical dendrite and the inner mitochondrial membrane. Corticospinal tract (CST) disintegration is one of the major clinical manifestations of HSP and is also observed in SPAST^C448Y^ mice (Qiang et al., 2019). In addition, ER fragmentation together with mitochondrial disintegration are reported in HSP patients and in mouse models of spastin HSP (Park et al., 2010; Sonda et al., 2021; Zhu et al., 2022). Therefore, we decided to investigate whether **NU-9** treatment would also have a beneficial impact on CSMN that degenerate due to the SPAST^C448Y^ mutation, in vivo.

Following 100-day treatment of **NU-9** (100 mg/kg/day dosage by gavage, 5 days a week, starting at P100), we observed significant improvement in the integrity and stability of corticospinal tract axon fibers. The **NU-9** treatment helped resolve the electron-dense accumulations in the soma, and it also improved mitochondria and endoplasmic reticulum (ER) stability. Interestingly, **NU-9** was reported to help CSMN overcome similar cellular challenges when they are diseased due to misfolded SOD1 toxicity and TDP-43 pathology (Genc et al., 2021).

Our findings not only lay a strong foundation for a clear understanding of the cellular and molecular basis of selective UMN vulnerability with respect to the SPAST^C448Y^ mutation, but also help develop a mechanism-focused drug discovery effort, in which compounds that prevent common cellular pathological events are identified and further developed to help neurons that display selective vulnerability, albeit at different diseases. Preclinical studies indicate that **NU-9** treatment reduces corticospinal axon degeneration and improves stability and integrity of key organelles in the CSMN of SPAST^C448Y^ mice. These results open the door for future and more detailed studies investigating functional improvements and effective translational efforts.

## 2. Materials and methods

### 2.1. Generation of SPAST^C448Y^-UeGFP reporter mice

All animal procedures were approved by the Northwestern University Animal Care and Use Committee and comply with the standards of the National Institutes of Health. All mice were in C57BL/6J background. UCHL1-eGFP mice were generated in the Ozdinler Laboratory (Yasvoina et al., 2013)(Jackson Laboratory (#: 022476). SPAST^C448Y^ mouse model of HSP is generated based on the mutation detected in the HSP patients (Qiang et al., 2019). Hemizygous UCHL1-eGFP females were mated with hemizygous SPAST^C448Y^ mice to generate SPAST^C448Y^-UeGFP mice, the UMN reporter line of the SPAST^C448Y^ model of HSP. Since behavioral deficits were reported to be stronger and more penetrant in males compared to females of SPAST^C448Y^ mice (Qiang et al., 2019), only male mice were used in this study.

### 2.2. NU-9 treatment by gavage

**NU-9** was generated in the Silverman lab based on its ability to reduce misfolded SOD1 aggregates (Benmohamed et al., 2011). Mice were weighed and a 100 mg/kg **NU-9** dosage was calculated and administered 5 days a week (Monday through Friday) by oral gavage, starting at postnatal day (P)100 and continuing until P200. SPAST^C448Y^-UeGFP (*n* = 4) and UeGFP mice (n = 4) were used for CSMN FACS purification and bottom-up proteomic studies. SPAST^C448Y^ with vehicle (*n* = 3), SPAST^C448Y^ with **NU-9** treatment (n = 4), and WT (n = 3) mice used for EM analyses. For oral administration, 20ga × 38 mm plastic feeding tubes were used (Instech Laboratories, Inc). The gavage tip was inserted into the mouth over the tongue and into the pharynx. The tip was safely and smoothly slid into the esophagus, while observing the swallowing reflex. Upon administration, the gavage tip was gently pulled out.

### 2.3. FACS purification of CSMN

Motor cortex from WT-UeGFP mice (healthy control), and SPAST^C448Y^-UeGFP mice (diseased) were microdissected at P60 and P90, using a fluorescence-equipped dissecting microscope (SMZ-1500; Nikon) in the presence of cold dissociation medium (20 mM glucose, 0.8 mM kynurenic acid, 0.05 mM D(− )-2-amino-5-phosphonovaleric acid (AP5), 50 U/ml penicillin, 0.05 mg/ml streptomycin, 0.9 M Na2SO_4_ and 0.014 M MgCl_2_, pH = 7.35, and supplemented with B27) and enzymatically digested for 15–20 min (0.16 mg/l L-cysteine HCl, 12 U/ml papain and 1 U/ml DNAseI, pH = 7.35, prepared in dissociation medium) at 37 ^◦^C. Enzymatic digestion was blocked by adding dissociation medium containing 10 mg/ml ovomucoid (Sigma) and 10 mg/ml bovine serum albumin (BSA). Cells were mechanically dissociated in trituration buffer (OPTIMEM, supplemented with 20 mM glucose, 0.4 mM kynurenic acid, 0.025 mM AP5, B27, and BSA). The supernatant was collected in trituration buffer for FACS purification using a 4 laser FACSAria cell sorter (Becton Dickinson). Cells were collected in PBS with 1× Halt-protease inhibitor and debris was excluded using SSC-A and FSC-A, FITC-A was used to identify CSMN. Dissociation and FACS purification yielded approximately 30,000 live CSMN per motor cortex, per mouse. Each experiment was repeated at least 4–5 times.

### 2.4. Bottom-up proteomic analyses

Proteins from each sample (*n* = 4 per group per genotype, a total of 16 samples) were purified by acetone/trichloroacetic acid precipitation. After pelleting and washing with ice-cold acetone, the resulting protein pellet was resuspended in 8 M urea and 0.4 M ammonium bicarbonate, reduced with 4 mM dithiothreitol, and alkylated with 18 mM iodoacetamide. The solution was then diluted to <2 M urea and 1 μg of trypsin was added prior to an overnight incubation at 37 ^◦^C. The resulting digested peptides were desalted using solid-phase extraction on the C18 spin column and the eluates were dried under a SpeedVac vacuum concentrator. Dried peptides were reconstituted in 0.1% formic acid, and concentrations were measured using a NanoDrop assay. Peptides were loaded onto a Neo trap cartridge coupled with an μPAC Neo High Throughput analytical C18 column (75 μm × 5.5 cm) using a Vanquish Neo UHPLC System. A 7-min gradient was applied from 3.2% to 36% solvent B (0.1% formic acid in acetonitrile) against solvent A (0.1% formic acid in water).

Mass spectrometry analysis was performed using an Orbitrap Astral Mass Spectrometer with a FAIMS Pro Duo interface (Thermo Fisher Scientific). Full scan acquisition was conducted at a resolution of 240 K with an AGC target of 500% and a maximum injection time of 3 msec. For DIA acquisition, the precursor range was set to 380–980 *m*/*z* with a 2 m/z isolation window. MS2 fragmentation was performed using 25% HCD collision energy, with an AGC target of 500% and a maximum injection time of 3 msec. The scan cycle was controlled with a loop time of 0.6 s, and the FAIMS compensation voltage was maintained at −45 V for both full scan and DIA. Data processing was carried out using Spectronaut 18 (Biognosys AG). Raw data files were analyzed via direct DIA against the mouse SwissProt database. Search parameters included trypsin digestion with cleavage after K or R (except when followed by P), allowance for up to two missed cleavages, carbamidomethylation of cysteine (static modification), and variable modifications including oxidized methionine and N-terminal acetylation. Peptide spectrum matches (PSMs), peptides, and protein groups were identified using a false discovery rate (FDR) threshold of 0.01. For P60, a total of 5772 proteins, and for P90 a total of 5407 proteins were detected (Supplemental Table 1).

All proteomic datasets have been submitted to the ProteomeXchange Consortium via the MassIVE repository (Accession #: PXD079642).

### 2.5. Immunocytochemistry

Upon completion of bottom-up proteomics, a set of proteins were highlighted to be expressed at higher or lower levels in pure populations of CSMN. The presence of these proteins were confirmed by immunocytochemistry using antibodies to Aldehyde Dehydrogenase 3 Family Member A1 (ALDH3A1) (Thermo Fisher), Nuclear Receptor Coactivator 2 (NCOA2) (Thermo Fisher), ATP synthase mitochondrial F1 complex assembly factor 2 (ATPAF2) (MyBioSource), Calcium Voltage-Gated Channel Auxiliary Subunit Gamma 7 (CACNG7) (MyBioSource), all in 1:500 dilution. CSMN were labeled with GFP (Abcam; 1:500 dilution). Appropriate secondary Alexa Fluor 488 and Alexa Fluor 555 antibodies (Invitrogen) were used at 1:1000 dilution.

### 2.6. Electron microscopy and data analyses

Coronal sections of brain spanning motor cortex and lumbar spinal cord sections were fixed with 4% PFA and 2% glutaraldehyde for 2 h at room temperature. To label UMNs in motor cortex, Ctip2 Immunostaining was performed (1:500, Sigma), and sections were developed with DAB (Vector Sciences). Spinal cord sections were not subjected to immunostaining as dorsal funiculus can be identified by its location. Sections were washed with 0.12 M phosphate buffer pH 7.4 and then treated with 1% osmium tetroxide in 0.12 M phosphate buffer pH 7.4 for 1 h at room temperature. Sections were washed three times with 0.12 M phosphate buffer followed by treatment with 10% UranyLess (Electron Microscopy Sciences) for 1 h at room temperature. Sections were dehydrated by an ascending series of alcohol concentrations (50, 70, 80, 90, and 100%) and propylene oxide (Electron Microscopy Sciences) followed by treatment with epoxy resin for 24 h on a shaker. Sections were treated with resin for 7 h at room temperature followed by flattening between transparency sheets and cured in an oven at 65 ^◦^C for 3 days. Motor cortex from brain and dorsal funiculus from spinal cord sections were carefully dissected out, mounted on resin blocks and cured in an oven at 65 ^◦^C for 3 days. The blocks were trimmed before proceeding for semi-thin and ultra-thin sectioning. Resin blocks were sectioned on a Leica Ultracut UC6 ultramicrotome (Leica Inc., Nussloch, Germany). Sections (70 nm) were collected on 200 mesh copper‑palladium grids. Ultra-thin sections were counterstained on a drop of UranyLess solution (Electron Microscopy Sciences) and 0.2% lead citrate (Electron Microscopy Sciences). Grids were examined on FEI Tecnai Spirit G2 TEM (FEI Company, Hillsboro, OR) and digital images were captured on an FEI Eagle camera. A total number of 81 CSMN (WT = 20; SPAST^C448Y^ + vehicle: 31; SPAST^C448Y^ + **NU-9**: 30) were quantitatively assessed. Mitochondria (WT = 495; SPAST^C448Y^ + vehicle: 771; SPAST^C448Y^ + **NU-9**: 950), ER cisternae (WT = 412; SPAST^C448Y^ + vehicle: 241; SPAST^C448Y^ + **NU-9**: 771), and electron-dense aggregates (WT = 9; SPAST^C448Y^ + vehicle: 179; SPAST^C448Y^ + **NU-9**: 81) were counted.

Five to ten EM images (2900×) were obtained from the corticospinal tract of each mouse (a total of 75 images from WT: *n* = 3 mouse; SPAST^C448Y^ + vehicle: n = 3 mouse; SPAST^C448Y^ + **NU-9:** *n* = 4 mouse). Images were in 8 bits, and the scale set by calibrating the image using a to 2 μm scale bar. A total of 5068 axon fibers were counted (WT: 2516; SPAST^C448Y^ + vehicle: 680; SPAST^C448Y^ + **NU-9**: 1872). The percentage of defective axon fibers were assessed and represented as per mouse for statistical analyses. Axon diameters were measured by drawing a line across the longest axis of each individual axon using the “straight line” tool of Image J. Quantification analysis was done using the Fereťs diameter function of *Image J*. The percent distribution of axons based on their diameter was prepared with 0.5 μm increments. The researcher performing data collection from EM images was blinded to experimental groups and genotypes.

### 2.7. Canonical pathway analyses

Proteins present in CSMN of SPAST^C448Y^-UeGFP mice that are significantly different from that of WT-UeGFP mice (*p <* 0.05; student *t*-test) were noted for P60 (*n* = 183; Supplemental Table 2) and P90 (*n* = 176; Supplemental Table 3). These proteins were uploaded to ingenuity pathway analyses (IPA; QIAGEN Comp, LA, USA). IPA is a toolbox that incorporates previously published knowledge domain for unbiased large data analysis. To reduce false-positive, we utilized very stringent inclusion criteria, such that only findings that are experimentally observed for direct binding with a published report and obtained from neurons and mammalian systems were included. Results obtained from non-mammalian systems, cancer cell lines, proteins that do not directly interact and curated findings were excluded. Only results with a published record of experimentally observed findings were included in the analyses. IPA was used to highlight canonical pathways the selected proteins are mostly involved in, the downstream regulators, upstream effectors as well as the interaction networks of uploaded proteins within given cellular events and pathways, as previously reported (Dervishi et al., 2018; Helmold et al., 2025; Helmold et al., 2023).

### 2.8. Statistical analysis

All statistical analyses were performed using Prism software (version 5.0a; Graphpad Software Inc., La Jolla, CA, USA). Proteins that are selected based on their expression levels in diseased and healthy CSMN at two different time points were analyzed using ordinary 2-way ANOVA with post hoc Tukey's multiple comparison test. The age, genotype and interaction were chosen as potential sources of variation to have the most conservative statistical output. Mitochondria, ER, and CST data were analyzed using ordinary 1-way ANOVA with post hoc Tukey's multiple comparison test. Data is presented as mean ± SEM because mean of each experimental group is compared with the mean of each other 3 experimental groups; *p < 0.05; **p < 0.01; ***p < 0.001; ****p < 0.0001.

## 3. Results

### 3.1. Protein content of CSMN in SPASTC448Y mice are dynamic

**Fig. 1.**
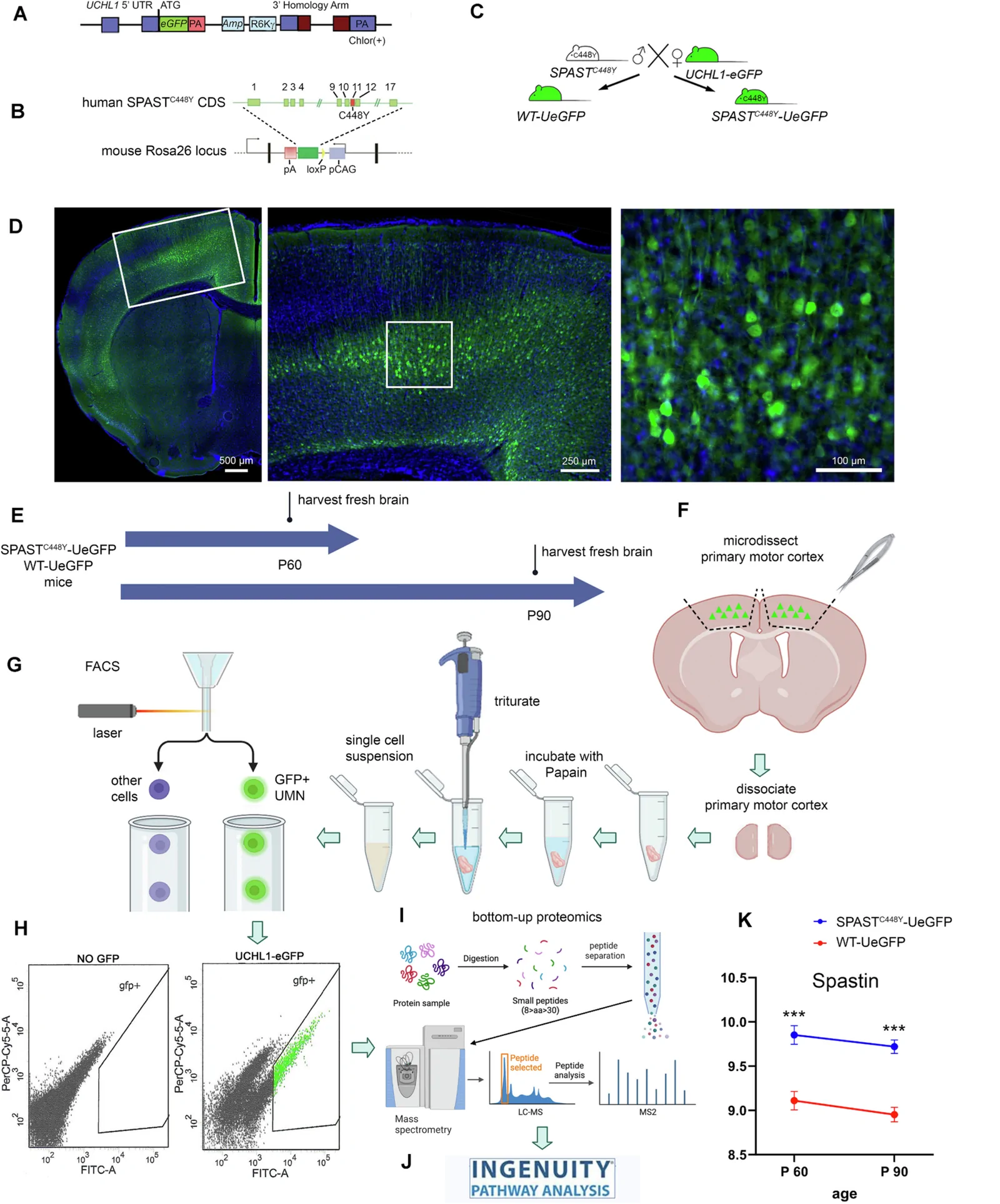
CSMN are made fluorescent in Spastin^C448Y^ background and are FACS-purified prior to bottom-up proteomic analyses, revealing cell type-specific changes in their proteome at P60 and P90. (**A-D**) Schematic drawing of the transgenic description of UCHL1-eGFP (**A**) and SPAST^C448Y^ (**B**) mice, and the mating strategy to generate WT-UeGFP (healthy control) and SPAST^C448Y^-UeGFP (diseased) mice (**C**), in which CSMN are eGFP+ (green) (**D**). (**E**) Schematic drawing of experimental design. Brain is isolated at P60 and P90 from WT-UeGFP and SPAST^C448Y^-UeGFP mice. (**F)** Motor cortex is isolated by microdissection and single cell suspension is prepared. (**G-H)** Pure populations of CSMN is collected by fluorescent activated cell sorting (FACS) and by setting proper gates using forward and side scatter and fluorescent intensity measures. (**I**) Pure populations of CSMN isolated from WT-UeGFP and SPAST^C448Y^-UeGFP mice are subjected to bottom-up proteomics. Proteins are further investigated by ingenuity pathway analyses (**J**). (**K**) Spastin protein levels are significantly higher in the SPAST^C448Y^-UeGFP mice, compared to WT-UeGFP mice at both ages. (For interpretation of the references to colour in this figure legend, the reader is referred to the web version of this article.)

To purify CSMN that become diseased due to an HSP-linked mutation in the *SPAST* gene, we crossed UCHL1-eGFP and SPAST^C448Y^ mice to generate WT-UeGFP (healthy control) and SPAST^C448Y^-UeGFP (diseased) mice (Fig. 1A-C). CSMN are in layer 5 of the motor cortex and express eGFP (Fig. 1D). CSMN identity of eGFP+ neurons was previously confirmed (Yasvoina et al., 2013), allowing their detailed cellular analyses at P60 and P90 (Fig. 1E). The primary motor cortex is microdissected out from both hemispheres (Fig. 1F). After single-cell suspension was prepared by enzymatic digestion and trituration, CSMN were isolated from both healthy (WT-UeGFP; *n* = 4) and diseased (SPAST^C448Y^-UeGFP; n = 4) mice via FACS purification (Fig. 1G), based on size, forward and side-scatter properties, and levels of green fluorescence (Fig. 1H). FACS-purified CSMN were further analyzed by bottom-up proteomics (Fig. 1I), and proteins that are significantly different between healthy and diseased CSMN at P60 (Supplemental Table 2) and at P90 (Supplemental Table 3) were further assessed by ingenuity pathway analyses (IPA). As expected, the levels of spastin protein were significantly higher in CSMN of SPAST^C448Y^-UeGFP mice at P60 (healthy: 9.11 ± 0.21; diseased: 9.85 ± 0.21; *p*-value:0.0024) and P90 (healthy: 8.95 ± 0.16; diseased: 9.72 ± 0.15; F (1,12) = 15.52, *p* = 0.00045, Fig. 1K).

**Fig. 2.**
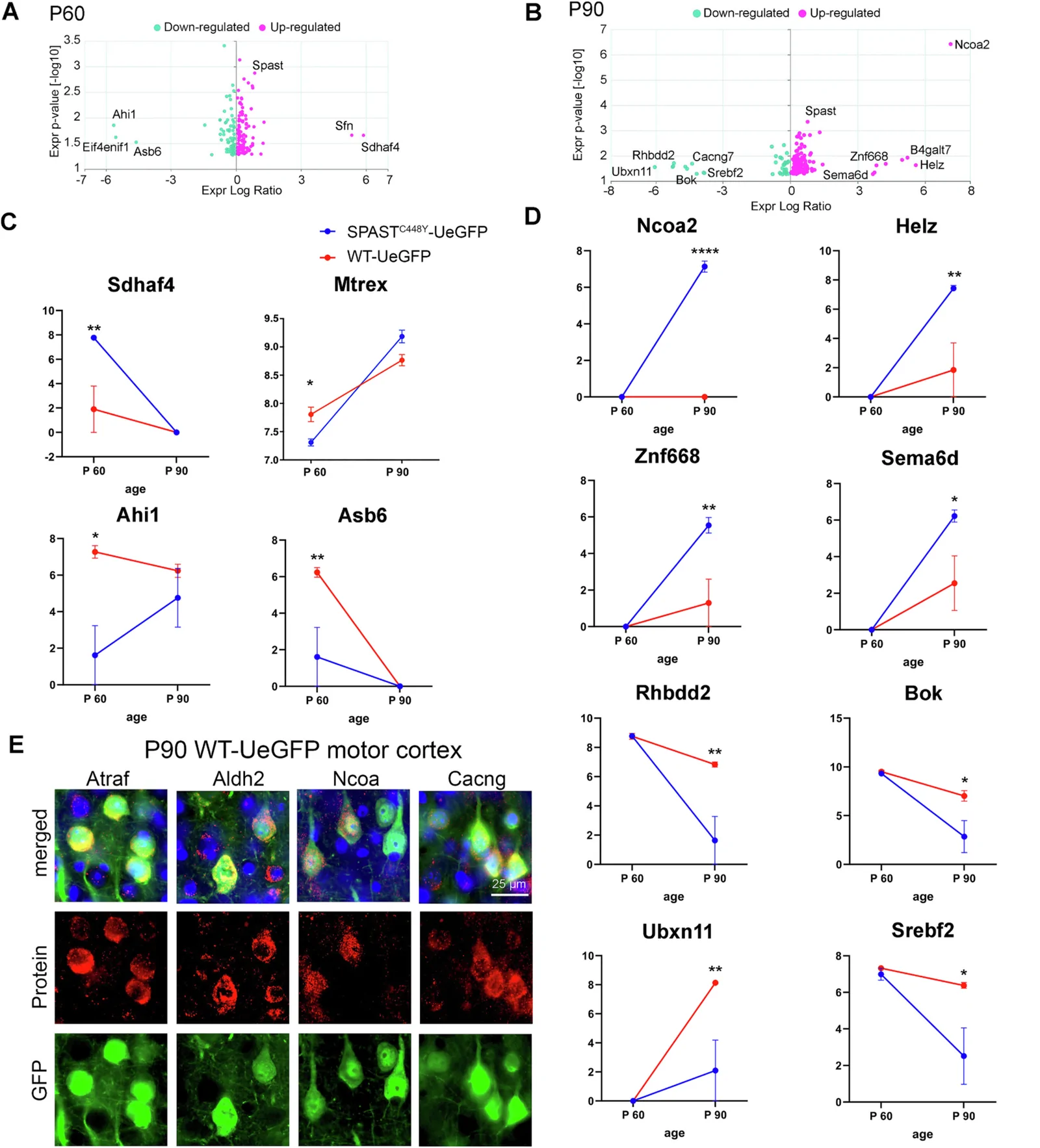
The protein profile of diseased CSMN is very dynamic. (**A-B**) The volcano-plot depicting proteins that are upregulated (pink) and downregulated (green) in CSMN isolated from SPAST^C448Y^-UeGFP mice in comparison to CSMN isolated from WT-UeGFP mice at P60 (**A**) and at P90 (**B**). (**C**) Line graphs of Sdhaf4, Mtrex, Ahi1, and Asb6, as examples of proteins that are significantly present in CSMN of SPAST^C448Y^-UeGFP mice at P60, but are comparable to that of WT-UeGFP mice at P90. (**D**) Line graphs of Ncoa2, Helz, Znf668, Sema6d, Rhbdd2, Bok, Ubxn11, and Srebf2, as examples of proteins that are significantly different in CSMN of SPAST^C448Y^-UeGFP mice at P90, but are comparable to that of WT-UeGFP mice at P60. (**E**) Images of CSMN (green) that express Atpaf, Aldh2, Ncoa, Cacng, proteins that are detected by bottom-up proteomic analyses. Top panel show merged image. Line graphs represent the mean of samples from *n* = 4 mice ±SEM. The asterisks represent the difference in significance: *p = 0.02, **p = 0.005, ****p = 0.0001 comparing mean of each group with each other using two-way ANOVA followed by Tukey's multiple comparison test. Scale bar: 25 μm. (For interpretation of the references to colour in this figure legend, the reader is referred to the web version of this article.)

Proteomic analyses revealed a list of proteins that are differentially present in pure populations of diseased CSMN at P60 (Fig. 2A; Supplemental Table 2) and P90 (Fig. 2B; Supplemental Table 3). At P60, a total of 183 proteins (cytoplasm (*n* = 110), nucleus (*n* = 29), membrane-bound (*n* = 24) and secreted proteins (*n* = 5)) showed significant difference between diseased and healthy CSMN. By P90, a total of 176 proteins (cytoplasm (*n* = 89), nucleus (n = 29), membrane (*n* = 39) and secreted proteins (*n* = 6)) were significantly different.

We found a dynamic change in the presence of key proteins in pure populations of CSMN. For example, Sdhaf4 (F (1,12) = 9.423, *p* = 0.0097) was higher in diseased CSMN at P60, but dropped to comparable levels by P90, whereas Mtrex (F(1,12) = 190.2, *p <* 0.0001), Ahi1 (F(1,12) =9.37, *p* = 0.0099) and Asb6 (F(1,12) = 7.99, *p* = 0.0152) were significantly lower at P60 but became comparable by P90 (Fig. 2C). On the other hand, Ncoa2 (F(1,12) = 554.2, p < 0.0001), Helz (F(1, 12) = 9.051, *p* = 0.0109)), Znf668 (F (1,12) = 9.632, *p* = 0.0091)), and Sema6d (F(1,12) = 5.762, *p* = 0.0335) were significantly higher at P90, while they were not detectable at P60 (Fig. 2D). In contrast, Rhbdd2 (F (1,12) = 9.583, *p* = 0.0093), Bok (F(1,12) = 6.229, *p* = 0.0281), Ubxn11 (F(1, 12) = 8.278, *p* = 0.0139), and Srebf2 (F (1,12) =7.014, *p* = 0.0212) were significantly reduced in the diseased CSMN at P90, while they were comparable at P60, further implicating a dynamic change in gene expression profiles of key proteins with respect to disease progression. (Please refer to Supplemental Table 4 for all individual data points for each time point and genotype).

Interestingly, about 93% of proteins did not overlap between P60 and P90 and showed a distinct profile at two different time points, suggesting the dynamic nature of cellular events that happen in CSMN prior to symptom onset. Immunocytochemistry analyses further confirmed expression profiles of proteins, such as Atraf, Aldh2, Ncoa, and Cacng in CSMN, in an intact brain (Fig. 2E).

**Fig. 3.**
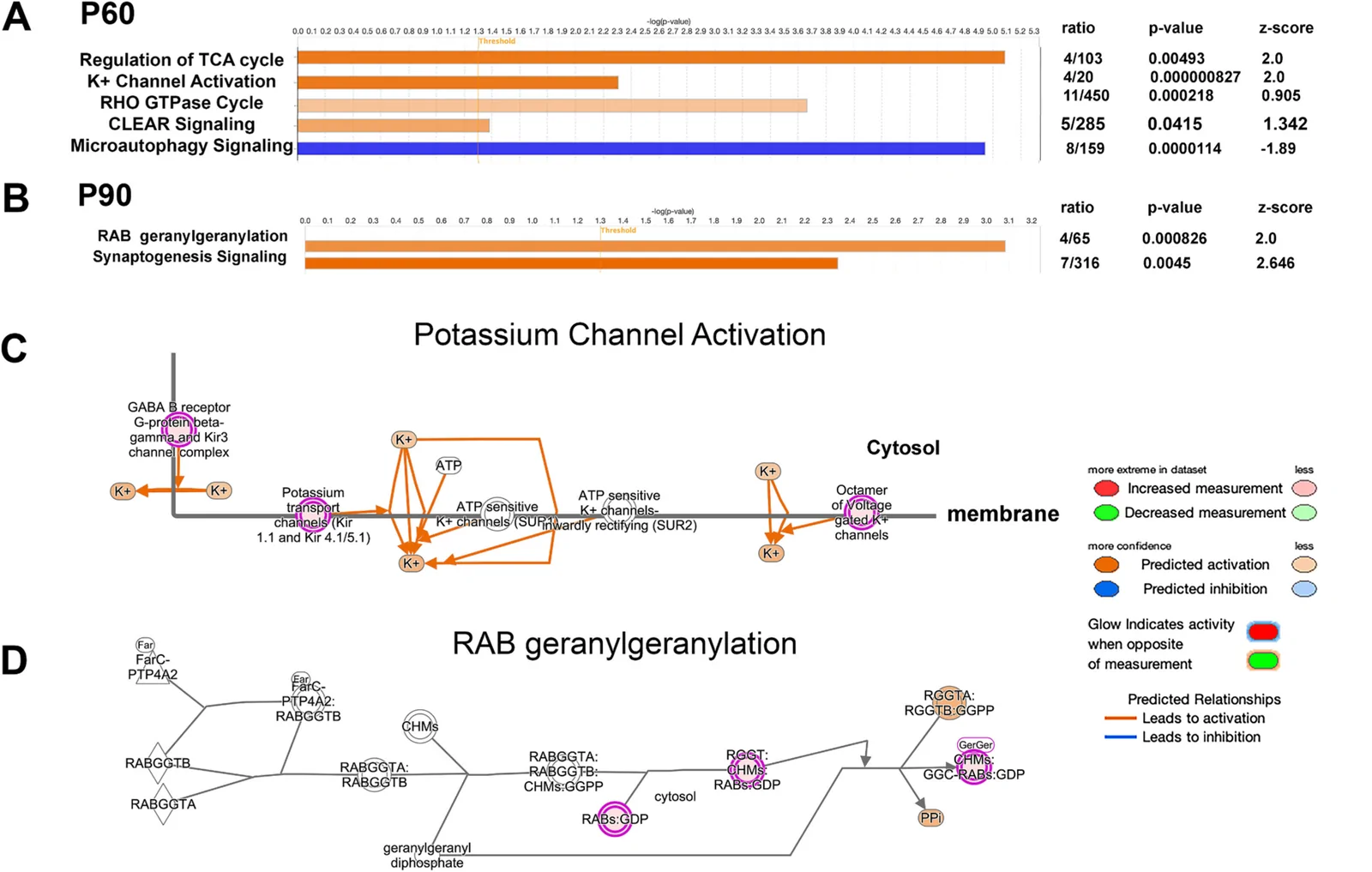
Key Canonical pathways that are active in diseased CSMN at P60 and P90. (**A-B**) A distinct set of canonical pathways were highlighted to be increasingly involved or reduced at P60 (**A**) and are present at P90 (**B**). The ratio, the *p*-value and the z-score is included to the right of each canonical pathway. (**C**) Schematic representation of the Potassium channel activation, depicting the segments of the pathway suggested to be upregulated based on changes detected in the presence of proteins. (**D**) Schematic representation of the RAB geranylgeranylation pathway, depicting the molecules and proteins within the pathway that are upregulated in diseased CSMN at P90.

To further understand cellular events that are primarily associated with the proteome changes, we performed canonical pathway analyses, using IPA. At P60, the voltage-gated potassium channels, namely, Kcnab2, Kcnd3, Kcnj10, and Kcnq5, were upregulated in the diseased CSMN, which was not observed at P90 (Supplemental Fig. 1). Therefore, a potential alteration in the voltage-gated potassium channel activity was highlighted (ratio: 4/103; *p*-value: 4.93 × 10^−3^; z-score: 2; Fig. 3 A, C). Proteins that are involved in ensuring proper execution of the Kreb's cycle in the mitochondria, namely Sdhaf4, Nfs1, Iscu, and Fxn, displayed increased expression profile in the diseased CSMN, suggesting an attempt to upregulate the tricarboxylic acid (TCA) cycle (ratio: 4/20; *p*-value: 8.27 × 10^−7^; z-score: 2). Interestingly, cellular events that are related to Rho GTPases were highlighted to be relevant (ratio: 11/450; p-value: 2.18 × 10^−4^, z-score: 2), with some proteins showing increased levels of expression (i.e., Ankle2, Arhgap35, Diaph1, Almo2, Hsp90aa1, Hspe1, and Stip1) and some showing reduced levels of expression (Arhgef2, Rnf20,Trio, and Wdr91). The CLEAR signaling was also detected to be involved at P60, with Prkab1 and Wdr45 upregulated, and Lamtor2 and stratifin (SFN) downregulated (ratio: 5/285; *p*-value: 4.15 × 10–2, z-score: 1.342; Fig. 3A). To the contrary, a potential decrease in the microautophagy signaling pathway was detected at this very early non-symptomatic age (ratio:8/159; *p*-value: 1.14 × 10^−5^; z-score: 1.89), because some key proteins that were expected to be at high expression levels were found to be reduced in the diseased CSMN, such as Psmd2, Psmd3, Psmd12, Psmd13, Sec13, and Vps37c (Fig. 3A; Supplemental Fig. 1).

The canonical pathways that were highlighted at P90, were rather different. At this age, RAB geranylgeranylation (ratio: 4/65; *p*-value: 8.26 × 10^−4^; z-score: 2; Fig. 3 B, D) was found to be one of the most relevant cellular pathways, with increased expression levels of Rab2a, Rab4b, Rab5b, and Rab5c proteins. In addition, the synaptogenesis signaling pathway was highlighted (ratio: 7/316; *p*-value: 4.5 × 10^−3^; z-score: 2.646), with Efnb3, Lyn, Plcg1, Rab5b, Rab5c, Rras, and Syt12 proteins involved in this pathway displayed an increased expression profile in diseased CSMN (Supplemental Fig. 2).

**Fig. 4.**
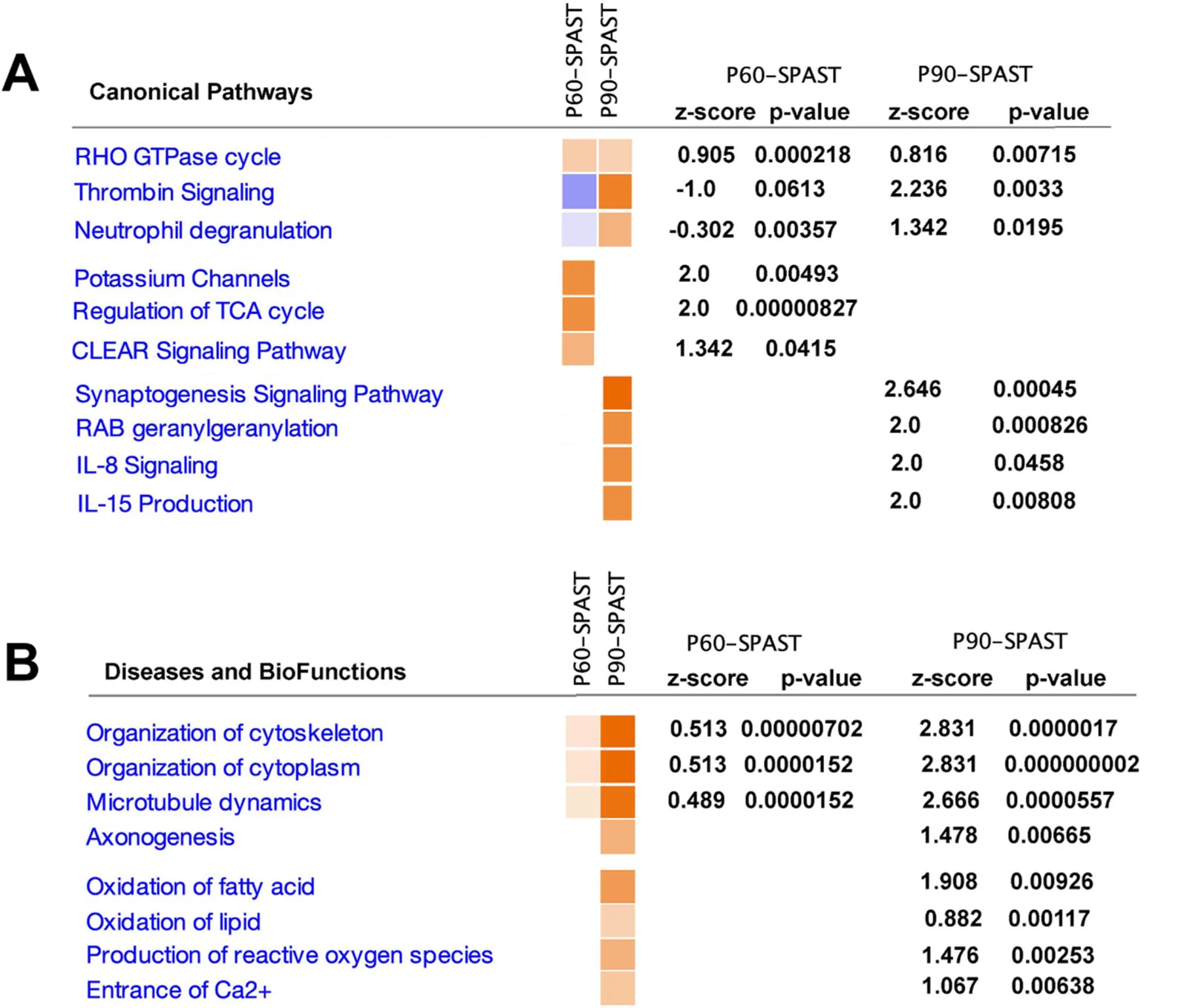
Canonical Pathways, Diseases and Biofunctions involved in diseased CSMN at P60 and P90. (**A**) Canonical pathways that are active in diseased CSMN at both time points or are present either at P60 or P90. (**B**) The disease related biofunctions that are highlighted to be present at both time points or beginning to occur at P90. *Z*-scores and *p*-values for each canonical pathway or biofunction mark relative significance in comparison to healthy CSMN.

We next wanted to compare the proteins detected at P60 and P90 and the extent of their involvement in previously established canonical pathways, diseases, and cellular functions. Interestingly, the RHO GTPase cycle was relevant at both ages (P60: z-score: 0.905; *p*-value: 2.18 × 10^−4^; P90: z-score: 0.816; *p*-value: 7.15 × 10^−3^, Fig. 4A). Also, even though the thrombin signaling was present at both ages, it was with a different connotation. For example, it was dysregulated, inhibited, or negatively associated at P60, but its intensity and relevance increased by P90 (P60: z-score: − 1, *p*-value: 0.0613; P90: z-score: 2.236, p-value: 0.0033, Fig. 4A). The same was true for neutrophil degranulation (P60: z-score: − 0.302, p-value: 0.00357; P90: z-score: 1.342, p-value: 0.0195, Fig. 4A), as its presence and relevance increased with age. There were also canonical pathways that were present either at P60 or at P90. For example, in addition to regulation of potassium channels and the TCA cycle, the CLEAR signaling pathway emerged to be one of the significant cellular events that happen at P60 (z-score: 1.342, p-value: 0.0415, Fig. 4A). At P90, IL-8 signaling (z-score: 2, p-value: 0.0458) and IL-15 signaling (z-score: 2, p-value: 0.00808) became apparent together with RAB geranylgeranylation (z-score: 2: p-value: 0.0008) and synaptogenesis signaling pathways (z-score: 2.646: p-value: 0.00045, Fig. 4A).

When disease related cellular functions were compared between the two time points, two key cellular events became apparent (Fig. 4B). One was related to the organization of the cytoplasm, and the other was related to the mitochondrial integrity and function. Changes associated with cytoarchitectural alterations were detected at P60, preceding the mitochondrial alterations that were instead detectable at P90. The efforts to regulate and organize the cytoskeleton (P60: z-score: 0.513, p-value: 7.02 × 10^−6^; P90: z-score: 2.831, p-value: 1.7 × 10^−6^), and the cytoplasm (P60: z-score: 0.513, p-value: 1.52 × 10–5; P90: z-score: 2.831, p-value: 2 × 10^−9^), were evident at P60 and increased by P90 (Fig. 4B). Likewise, the significant proteins, both at P60 and P90, were related to maintaining microtubule dynamics (P60: z-value: 0.489, p-value: 1.52 × 10–5). However, the effort to regenerate axons were detected (z-score: 1.478, p-value: 0.00665, Fig. 4B), supporting the idea that axonal deficiencies are early in the disease and that diseased CSMN attempt to restore CST at this early pre-symptomatic stage.

A second major cellular weakness was suggested to occur at the site of mitochondria and ER, with increased oxidation of fatty acids (P90: z-score: 1.908, p-value: 0.00926), oxidation of lipids (P90: z-score: 0.882, p-value: 0.00117), production of reactive oxygen species (P90: z-score: 1.476, p-value: 0.00253), and cellular entrance of Ca^+2^ (P90: z-score: 1.067, p-value: 0.00638, Fig. 4B). These cellular functions suggest potential limitations in both mitochondria and ER, which have been previously observed in HSP patients and models of HSP (Errico et al., 2002; Evans et al., 2005; Raby et al., 2024; Roll-Mecak and Vale, 2005; Vajente et al., 2019).

### 3.2. Electron microscopic analyses reveal cellular pathology, which correlates with changes detected by proteomics

**Fig. 5.**
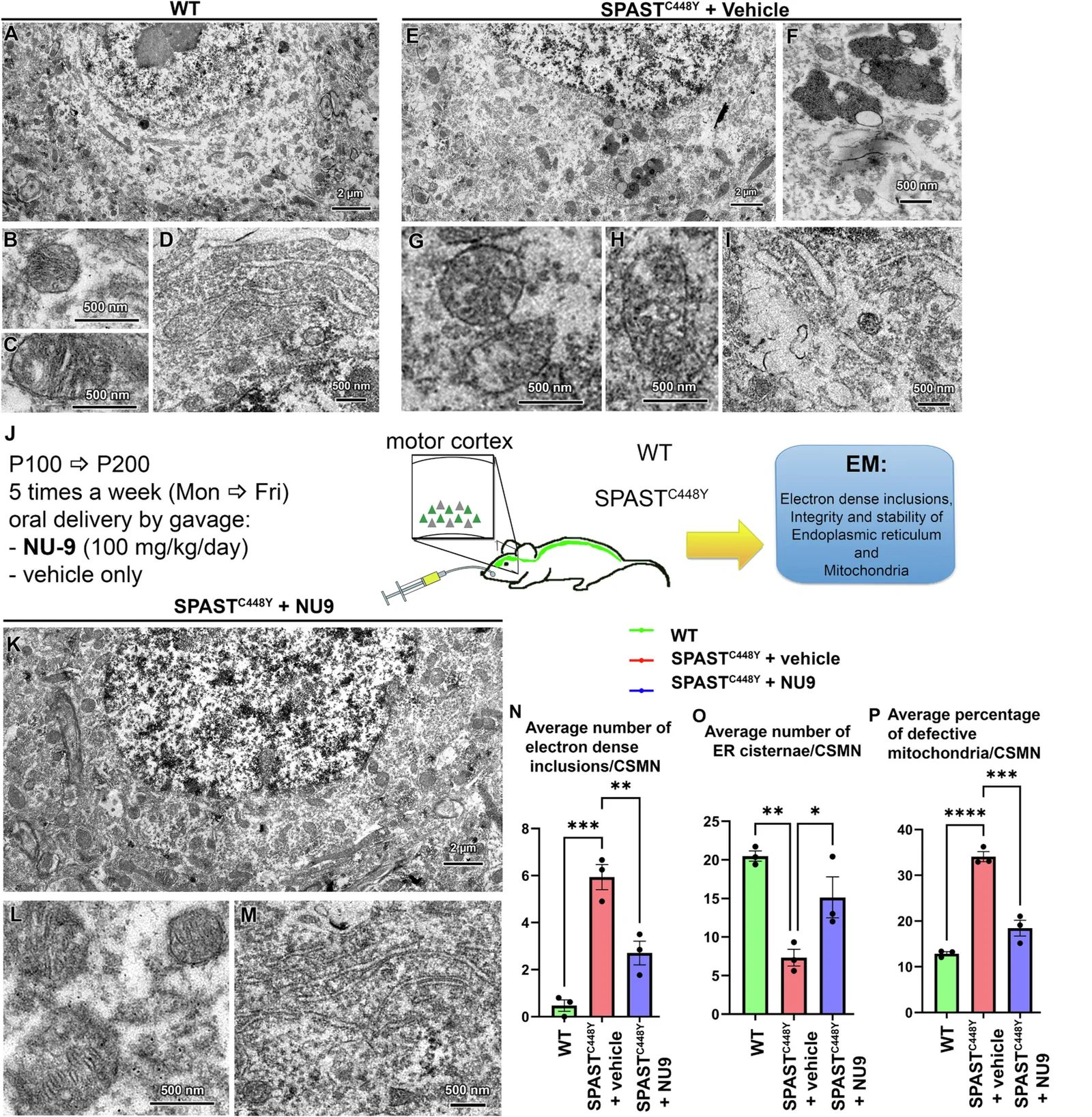
NU-9 Treatment Help Improve disease related cellular problems in CSMN of SPAST^C448Y^ mice. **(A)** A representative electron microscopic image of Ctip2+ healthy CSMN, mitochondria **(B, C)**, and endoplasmic reticulum (ER) **(D)** in CSMN of WT mouse. **(E)** A representative electron microscopic image of Ctip2+ diseased CSMN, with electron dense inclusions (**F**), mitochondrial defects **(G, H)**, and problems with ER **(I). (J)** Schematic representation of **NU-9** treatment strategy and experimental workflow. WT and SPAST^C448Y^ mice are treated with either **NU-9** (100 mg/kg/day for 5 days/week for 100 days) or with vehicle via gavage, starting at P100, ending at P200. CSMN is investigated with EM. **(K)** A representative electron microscopic image of Ctip2+ CSMN of SPAST^C448Y^ mice treated with **NU-9**, with improved mitochondria **(L)**, and ER **(M).** (**N**–**P**) Bar graph representation of quantification of average number of electron dense inclusion/CSMN (Number of mice: WT, *n* = 3; SPAST^C448Y^ + vehicle, *n* = 3; SPAST^C448Y^ + NU-9, *n* = 3). One-way ANOVA followed by Tukey's *post-hoc* test was performed: F (2, 6) = 37.98, *P* = 0.0004. Data were represented as ± SEM (**N**), average number of ER cisternae/CSMN (Number of mice: WT, *n* = 3; SPAST^C448Y^ + vehicle, *n* = 3; SPAST^C448Y^ + NU-9, *n* = 3). One-way ANOVA followed by Tukey's *post-hoc* test was performed: F (2, 6) = 15.19, *P* = 0.0045. Data were represented as ± SEM (**O**), and average number of defective mitochondria/CSMN (Number of mice: WT, *n* = 3; SPAST^C448Y^ + vehicle, *n* = 3; SPAST^C448Y^ + NU-9, *n* = 3). One-way ANOVA followed by Tukey's *post-hoc* test was performed: F (2, 6) = 83.19, *P* = 0.0001. Data were represented as ± SEM (**P**). Scale bar: 2 μm (A, E, K), 500 nm (B, C, D, F, G, H, I, L, and M). *p = 0.02, **p = 0.005, ***p = 0.0003, ****p = 0.0001.

**Fig. 6.**
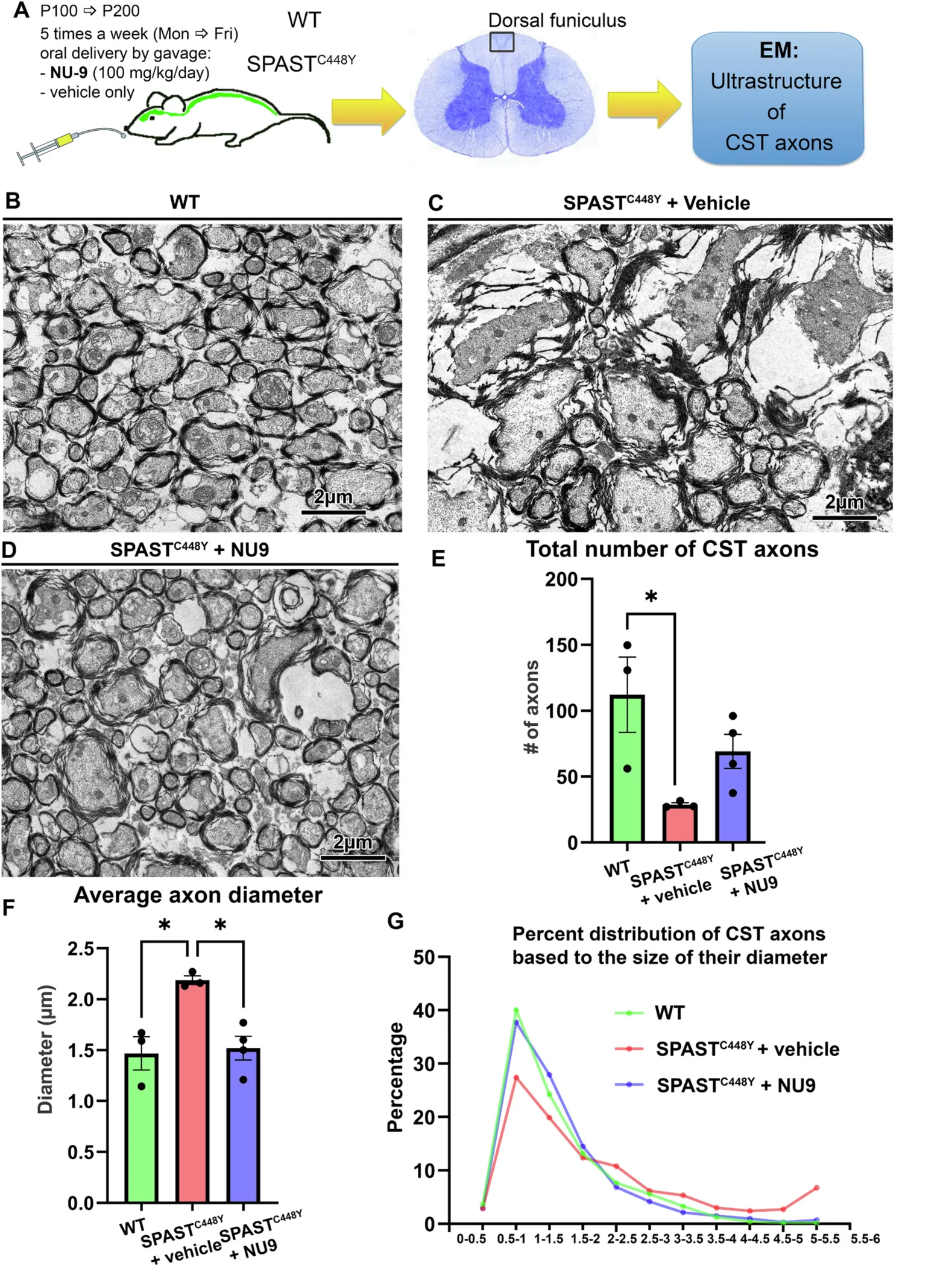
NU-9 treatment improves structural integrity of corticospinal tract axon. **(A)** Schematic representation of the experimental workflow. WT-UeGFP and SPAST^C448Y^-UeGFP mice are treated with either **NU-9** (100 mg/kg/day for 5 days/week) or vehicle. The dorsal funiculus was isolated and analyzed by EM. **(B)** A representative image of CST axons from WT-UeGFP mouse. **(C)** A representative image of CST axons from SPAST^C448Y^-UeGFP mouse treated with vehicle. **(D)** A representative image of CST axons from SPAST^C448Y^-UeGFP mouse treated with **NU-9**. **(E)** Bar graph representation for the quantification of total number of CST axons within the dorsal funiculus (Number of mice: WT, *n* = 3; SPAST^C448Y^ + vehicle, *n* = 3; SPAST^C448Y^ + NU-9, *n* = 4). One-way ANOVA followed by Tukey's *post-hoc* test was performed: F (2, 7) = 5.282, *P* = 0.04. Data were represented as ± SEM. **(F)** Bar graph representation for the quantification of average CST axon diameter (Number of mice: WT, *n* = 3; SPAST^C448Y^ + vehicle, *n* = 3; SPAST^C448Y^ + NU-9, *n* = 4). One-way ANOVA followed by Tukey's *post-hoc* test was performed: F (2, 7) = 10.40, *P* = 0.008. Data were represented as ± SEM. **(G)** Percent distribution of CST axon fibers based on the size of their diameter. Scale bar: 2 μm. *p = 0.02.

We utilized electron microscopy (EM) to visualize both somatic (Fig. 5) and axonal (Fig. 6) compartments of diseased CSMN, with respect to healthy neurons. When CSMN somata was investigated in detail, numerous electron-dense accumulations with different size and shape were detected in SPAST^C448Y^ mice (Fig. 5F), which were either absent or were rare in the CSMN of healthy mice. The mitochondria displayed damaged inner mitochondrial membrane (Fig. 5G-H), whereas the mitochondria in age-matched healthy CSMN were intact (Fig. 5B-C). In addition, integrity of the ER was compromised, such that the ER cisternae were shorter, broken, and swollen in diseased CSMN (Fig. 5I). Such cellular defects were not observed in healthy CSMN at P120 (Fig. 5D), suggesting that they are not primarily age-related.

### 3.3. NU-9 improves the underlying disease-causing factors in CSMN of SPASTC448Y-UeGFP mice

**NU-9** was first developed from initial hits that were able to reduce misfolded SOD1 aggregates (Benmohamed et al., 2011) and then was shown to improve the health of CSMN that degenerate due to misfolded SOD1 toxicity and TDP-43 pathology, in two different mouse models of ALS (Genc et al., 2021). Since **NU-9** treatment reduced protein aggregation, improved the integrity of mitochondria and ER upon in vivo treatment, and enhanced axon outgrowth in an in vitro study (Genc et al., 2022), and because similar cellular problems were observed in the CSMN of SPAST^C448Y^-UeGFP mice, we investigated whether **NU-9** treatment would also help reduce their burden. WT and SPAST^C448Y^ mice were treated with **NU-9** for 100 days, (100 mg/kg, 5 days/week via gavage) starting at P100 and ending at P200 (Fig. 5J).

We detected major improvements at the site of mitochondria (Fig. 5K, L) and ER (Fig. 5 M) and a significant reduction in the numbers of electron-dense inclusions following 100-day **NU-9** treatment (WT: 0.47 ± 0.24/CSMN, *n* = 20 CSMN; *n* = 3 mice; SPAST^C448Y^ + vehicle: 5.93 ± 0.53/CSMN, *n* = 31 CSMN; n = 3 mice, *p* = 0.003; SPAST^C448Y^ + **NU-9**: 2.70 ± 0.50/CSMN, *n* = 30 CSMN, *n* = 3 mice, *p* = 0.005, Fig. 5N).

The ER is a major site of protein synthesis with many other important cellular functions, such as maintaining lipid homeostasis, Ca^+2^ buffering, and cellular homeostasis. Diseased CSMN had many signs of disintegrating ER, with broken and shorter cisternae and the remaining ones were swollen and disintegrated. Healthy CSMN had intact ER cisternae (WT: 20.49 ± 0.66 cisternae/CSMN, n = 20 CSMN; n = 3 mice, Fig. 5D), whereas diseased CSMN treated with vehicle had mostly lost the integrity of the ER (SPAST^C448Y^ + vehicle: 7.31 ± 1.08 cisternae/CSMN, n = 31 CSMN; n = 3 mice, *p* = 0.003, Fig. 5I). **NU-9** treatment significantly increased the numbers of intact ER cisternae in diseased CSMN (SPAST^C448Y^ + **NU-9**: 15.14 ± 2.65 cisternae/CSMN, n = 30 CSMN; n = 3, *p* = 0.039, Fig. 5M).

CSMN that are diseased with misfolded SOD1 toxicity and TDP-43 pathology displayed mitochondrial disintegration early in the disease (Gautam et al., 2019b; Genc et al., 2021). Likewise, albeit the diseases are different and occur due to mutations in different genes, CSMN of SPAST^C448Y^ mice had comparable limitations (SPAST^C448Y^ + vehicle: 34.08 ± 1.08%, n = 31 CSMN; n = 3 mice, Fig. 5 G, H) when compared to healthy CSMN (WT: 12.89 ± 0.41%, n = 20 CSMN, n = 3; *p* = 0.0001, Fig. 5 B, C). Upon **NU-9** treatment, the percentage of impaired mitochondria in diseased CSMN were significantly reduced as more structurally intact mitochondria were detected in the soma (SPAST^C448Y^ + **NU-9**: 18.44 ± 1.73%, n = 30 CSMN; *n* = 3, *p* = 0.0002, Fig. 5 L, P).

HSP is mostly characterized by spasticity in patients, which is manifested mostly due to disintegrating CST axons (Blackstone, 2012; Blackstone, 2018; Fink, 2008). Therefore, it was important to investigate whether **NU-9** treatment would improve structural integrity of CST axons (Fig. 6A). We find that CST of diseased CSMN had significantly reduced numbers of healthy axons (SPAST^C448Y^ + vehicle: 29 ± 1.47; *n* = 26 images; n = 3 mice) when compared to that of healthy CSMN (WT: 112 ± 28.8; n = 20 images; n = 3 mice;,*p* = 0.033; Fig. 6 B-E). With **NU-9** treatment, however, the extent of CST axon degeneration was ameliorated, and axon fiber numbers became comparable to that of healthy cases (SPAST^C448Y^ + **NU-9**: 69 ± 12.9, *n* = 23 images; *n* = 4 mice, *p* = 0.276, Fig. 6E).

In addition, axonal swellings were observed in unhealthy and degenerating axons (Fig. 6C). CST axons of the Spastin^C448Y^-UeGFP mice also had increased swelling when compared to WT controls. (WT: 1.46 ± 0.16 μm, *n* = 2233 axon fibers counted in 20 images; n = 3 mice; SPAST^C448Y^ + vehicle: 2.18 ± 0.04 μm, *n* = 692 axon fibers counted in 26 images; n = 3 mice, *p* = 0.012, Fig.6 F, G). **NU-9** treatment significantly reduced swelling of CST axon fibers located in dorsal funiculus (SPAST^C448Y^ + **NU-9**: 1.52 ± 0.11 μm, *n* = 1911 axon fibers counted in 23 images, n = 4 mice, p = 0.012). We next investigated whether this improvement was restricted to a subset of axon fiber or whether there was a broader overall improvement. The percent distribution of CST axons based on the size of their diameter revealed that about 40% of axon fibers were within a 0.5–1 μm range, whereas this percentage was reduced to about 28% in diseased CSMN (Fig. 6G). About 10% of diseased CST axons had axon fibers that were about 5 μm in diameter, whereas almost none of the heathy CSMN had CST axons swollen as such. **NU-9** treatment shifted the curve in two ways: it increased the percentage of diseased CST axons that are not swollen and reduced the presence of swollen axons (Fig. 6G).

## 4. Discussion

The brain is complex and heterogenous. Understanding the cellular and molecular basis of selective neuronal vulnerability cannot be achieved by using homogenized brain tissue, and requires a direct and precise assessment of the neurons that display early vulnerability and progressive degeneration. The UMNs, which are known as Betz cells in humans and CSMN in mice, display selective vulnerability especially in UMN diseases. However, the underlying causes of their degeneration may differ. Therefore, focusing our attention to the neurons that degenerate becomes important and essential. Numerous “humanized” mouse models, which overexpress the mutated genes identified in affected patients, have been generated. SPAST^C448Y^ mouse model is based on the C448Y mutation detected in the *SPAST* gene in HSP patients, and mutations in this gene accounts for about 40% of all HSP cases (Qiang et al., 2019). When this well-characterized HSP model is crossed with UCHL1-eGFP mice, the CSMN become fluorescent within the disease background and their isolation via FACS-mediated approaches enable cellular and molecular assessment of their intrinsic and cell-type specific problems.

Proteomics offer great advantages for revealing the protein content of cells and the precision of the technology enable detection of even very small quantities of proteins. Therefore, we utilize quantitative bottom-up approaches to reveal the proteins present in pure populations of UMNs, and not in homogenized tissue extracts from the brain. Since there is selective neuronal vulnerability and not all neurons or cells display neurodegeneration to the same extent and with the same timing, and that UMNs are the vulnerable neuron populations in HSP, it was important to investigate UMNs as a pure neuron population.

We performed our studies at P60 and P90, to bring a temporal resolution at a time when SPAST^C448Y^ mice do not yet display motor function defects and can be considered in an “early presymptomatic stage”. Unbiased protein interaction network analyses begin to highlight cellular events that are most likely related to the identified proteins with a positive z score and *p*-value, and that their identified relationship cannot be explained by sheer luck. This large data-driven unbiased analyses approach has many advantages. Especially within the discovery mode, it lays out all potential cellular events that are suggested to be primarily associated with the given proteins without bias, but with p and z values that suggest significance and strong correlation.

Using unbiased proteomic data analyses, we find that the protein content of CSMN at P60 and P90 are very different. Even within 30 days, there was an immense change both in the numbers and the content of proteins that are present in diseased CSMN. These results highlight the dynamic nature of events that take place in vulnerable neurons as they progress more towards the diseased state. Interestingly, one of the cellular shortcomings was observed in the voltage-gated K^+^ channel activation (*p* = 0.005; z score: 2). Voltage-gated K+ channels are significantly important for reducing the risk of hyperexcitation (Joseph et al., 2025). Our data suggested upregulation of the Kir3 channel complex, which includes Kcnj3, Kcnj6, Kcnj9, and Kcnj5, in addition to Kir1.1 (Kcnj1) in CSMN that are at the initial stages of their vulnerability. Interestingly, unbiased large-data analyses suggest that Kcnj8 and Kcnj11 may be interacting together with Sur2, the regulatory subunit, to form a functional K-ATP channel, an ATP-sensitive potassium channel, which senses the energy state of the neuron and opens when ATP levels are low, so that the neuron becomes less excitable and save energy by not firing (Vidal-Taboada et al., 2018).

At this initial stage for CSMN vulnerability, four key cellular events begin to emerge: first is the regulation of TCA cycle, the second is regulation of Rho GTPase cycle, the third is CLEAR signaling pathway, and the fourth is reduction in the neuron's ability to clean itself from intercellular debris, the microautophagy pathway. Emergence of the TCA cycle as one of the canonical pathways that is altered suggests the neuron's inability to generate ATP, limitations in the energy pathways, and problems with mitochondria. This aligns with the changes in the voltage-gated K^+^ channel expression profile and suggests that ATP depletion could be an imminent problem. Reduced microautophagy, on the other hand, may suggest that emergence of electron-dense aggregates begin to occur prior to symptom onset and that dysregulated membrane dynamics could be a contributing factor (Sakai et al., 2026).

The Rho GTPase cycle is an important contributor to membrane fluidity and proper functioning, as they regulate the cytoskeleton and membrane-associated processes (Droppelmann et al., 2014), by serving as the link between actin cytoarchitecture modeling and membrane dynamics. RhoA, Rac1, and Cdc42 play significant roles in the formation of stress fibers, actin cytoskeleton remodeling, endocytosis, exocytosis, and vesicle formation, which are required for neuronal function (Bement et al., 2024), and their contribution to ALS pathology is beginning to emerge (Droppelmann et al., 2014). We find that disrupted Rho GTPase cycle is present both at P60 and P90, representing a persistent problem in HSP.

At P60, in addition to the voltage-gated K^+^-channel activation and regulation of the TCA cycle, as observed in Fig. 3, the CLEAR signaling pathway becomes detectable (z score: 1.342: *p*-value: 0.0415), which is one of the most important cellular pathways activated by increasing the gene expression profile of about 400 distinct genes that are important for maintaining cellular homeostasis (Franco-Juarez et al., 2022; Sardiello et al., 2009), most notably lysosomal biogenesis, autophagy, mitochondrial dysfunction, and cytoarchitectural stability (Palmieri et al., 2011). Recent evidence reveal that lysosomal dysfunction is one of the pathological features of spastin-HSP (Zhi et al., 2025; Zlamalova et al., 2024). CLEAR signaling pathway has been implicated in neurodegenerative diseases (Gu et al., 2022; Sardiello and Ballabio, 2009), and it is possible that vulnerable neurons activate this pathway to promote survival at this early stage.

By P90, the protein content of diseased UMNs greatly differ, further emphasizing the dynamic changes that occur in vulnerable neurons. At this age RAB geranylgeranylation and synaptogenesis signaling emerge as the key hurdles CSMN are faced with. RAB geranylgeranylation is a post-translational lipid modification, in which the geranylgeranyl groups are added to the Rab proteins, so that they can be associated with membranes via a hydrophobic anchor, required for maintaining membrane structure, fluidity, and function (Leung et al., 2006). Rab proteins are important in controlling vesicle dynamics by having a control over how they bud off, move or fuse (Homma et al., 2021; Stenmark, 2009). Interestingly, VPS37A (a.k.a. SPG53), which is involved in endosomal sorting and is part of the Rab geranylgeranylation pathway, is mutated in HSP patients (Zivony-Elboum et al., 2012), forming a direct genetic link to disease pathology.

Synaptogenesis signaling refers to the neurons' ability to communicate via synapses. This could be impaired when spines cannot be maintained, and the signaling is impaired. Wnt signaling and mTOR signaling are studied within the context of altered synaptogenesis signaling. Studies show alterations of Wnt signaling in the ALS brain (Gonzalez-Fernandez et al., 2020) and a clear association between autophagy and ALS (Chua et al., 2022). In addition, spine loss has been reported in diseased CSMN with TDP-43 pathology both in mouse models (Dyer et al., 2021; Fogarty et al., 2015) and patients (Genc et al., 2017). We also found that cortical connectivity alterations occur prior to symptom onset in the hSOD1^G93A^ model (Jara et al., 2020) and Alsin model of ALS (Dey et al., 2025).

IL-8 signaling (z score: 2.0: p-value: 0.046) and IL-15 (z score: 2.0: p-value: 0.008) production were also suggested to be upregulated based on the changes in protein profiles of CSMN isolated from diseased mice. IL-8 (CXCL8) is a chemokine that is known to recruit immune cells (Rusconi et al., 2017), and IL-15 is a cytokine that activates and maintains the natural killer cells and T-cells of the immune system and are increased in ALS patients' CSF (Rentzos et al., 2010). Emergence of these cytokines and chemokines at this presymptomatic stage suggest the initial phases of immune response could be activated in CSMN by P90, prior to disease onset.

The cellular problems gradually increase between P60 and P90, namely limitations with the organization of the cytoskeleton and microtubule dynamics, speak volumes to the inability of the diseased CSMN to maintain its cytoarchitecture in the absence of spastin function. Axonal defects become significant at P90, and EM analyses help visualize the extent of the CST axon damage. In addition, increased oxidation of fatty acids and lipids, together with the enhanced production of reactive oxygen species, implicate major limitations with mitochondrial function, which is mostly observed when the TCA cycle is impaired, and the neuron is trying to generate ATP by using long-chain fatty acids as an alternative source. In line with these findings, a dysregulation in Ca^+2^ levels is also suggested at P90. The cell-type specific alterations revealed by changes in proteins were confirmed by EM-level detailed cellular analyses, further highlighting key cellular events that are perturbed in diseased CSMN.

Interestingly, CSMN that are vulnerable to degeneration in the SPAST^C448Y^ mice showed many common features when compared to CSMN that degenerate due to misfolded SOD1 toxicity and TDP-43 pathology. Namely, the presence of electron dense accumulations and aggregates, mitochondrial and cytoarchitectural disintegrations were shared. These findings further emphasize the importance of studying diseases with cellular precision so that treatment strategies that focus on shared pathologies can be developed.

Since **NU-9** was identified to reduce protein aggregation, improve mitochondrial structure, and the integrity of ER in CSMN that are diseased due to misfolded SOD1 toxicity and TDP-43 pathology (Genc et al., 2021), and because similar cellular problems in the CSMN of SPAST^C448Y^-UeGFP mice were observed, we investigated whether **NU-9** treatment would also have a positive impact on improving the underlying causes of cellular degeneration within the context of HSP. Our findings reveal an immense potential for **NU-9** and its ability to improve the common underlying causes of neuronal vulnerability, that are shared among UMN diseases. **NU-9** treatment improved the integrity of CST axons, the stability of mitochondria and ER, and reduced the extent of electron-dense accumulations in the soma. These findings were comparable to that of CSMN in the SOD1^G93A^ mice and the TDP-43^A315T^ mouse model of ALS with TDP-43 pathology. Since SPAST^C448Y^-UeGFP mice develop the disease phenotype much slower and later than the SOD1^G93A^ mice and the TDP-43^A315T^ mouse, the time of **NU-9** exposure was longer in the SPAST^C448Y^-UeGFP mice by 40 more days, and the treatment was initiated at P100.

It is interesting that a small molecule would display similar outcomes in CSMN that degenerate in three different disease models. Further, **NU-9** was recently shown to reduce the levels of amyloid β, inside the vulnerable neurons of 5XFAD mouse model, a well-established Alzheimer's disease model (Kranz et al., 2025). Therefore, the evidence is consistent with **NU-9** preventing a subset of common underlying causes that are responsible for neuronal degeneration, albeit present in different and unrelated neurodegenerative diseases.

Since our investigation is based on phenotypic screening, observing the outcome of **NU-9** treatment falls short of identifying the binding partners of **NU-9** and explaining the mechanism of action. However, utilization of FACS purification to interrogate a specific neuron population, performing proteomics directly to them, confirming findings with EM, and therapeutic intervention with a compound that is proven to be affective in cellular problems that are shared among different diseases, substantially increases confidence in the biological conclusions. Alas, it is remarkable that a single small molecule, which received FDA approval to initiate Phase 1 clinical trials for ALS treatment, can also improve four major underlying causes of neuronal vulnerability in CSMN that are diseased due to a mutation in the SPAST gene.

Future studies with an increased cohort need to be performed to investigate whether changes in gene expression or protein dynamics would bring a mechanistic insight to our understanding of **NU-9**'s mode of action. It is also important to identify target engagement biomarkers and pharmacokinetic biomarkers that would inform about the timing and the extent of CSMN improvement upon **NU-9** treatment. We anticipate that mechanism-focused drug discovery efforts will take center-stage in the coming years, such that compounds that address common problems observed in different neurodegenerative diseases will be investigated not for one disease, but for their broader impact.

## Supplementary Material

Supplementary Table 1

Supplementary Table 2

Supplementary Table 3

Supplementary Table 4

Supp.Figure2

Supp.figure1

Supplementary data to this article can be found online at https://doi.org/10.1016/j.nbd.2026.107494.

All proteomic datasets have been submitted to the ProteomeXchange Consortium via the MassIVE repository (Accession #: PXD079642).

## Data Availability

Data will be made available on request.
